# Comparative efficacy of subsequent-line therapies for advanced triple-negative breast cancer: a bayesian network meta-analysis

**DOI:** 10.3389/or.2026.1834466

**Published:** 2026-07-09

**Authors:** Zheng Xu, Linxian Ding, Tai Zhao, Zhoumin Xu

**Affiliations:** 1 College of Mathematics, Beijing Normal University, Beijing, China; 2 Department of Oncology, Shidong Hospital Affiliated to University of Shanghai for Science and Technology, Shanghai, China

**Keywords:** antibody-drug conjugate, chemotherapy, network meta-analysis, objective response rate, progression-free survival, subsequent-line therapy, triple-negative breast cancer

## Abstract

**Background:**

Direct head-to-head randomized comparisons among emerging therapies are limited, making it difficult to determine the optimal subsequent-line treatment for advanced triple-negative breast cancer (TNBC). This study aimed to systematically compare the relative efficacy of second- and later-line regimens using a Bayesian network meta-analysis (NMA).

**Methods:**

PubMed, Embase, Web of Science, Cochrane Library and ClinicalTrials.gov were searched to 30 September 2025 for randomized controlled trials (RCTs) of second-/later-line advanced TNBC. A Bayesian NMA estimated effects on objective response rate (ORR), progression-free survival (PFS), and overall survival (OS). SUCRA was used for ranking, and model convergence was assessed by Gelman-Rubin diagnostics. Single-arm studies were included only in sensitivity analyses using a down-weighted binomial model for ORR. Additional sensitivity analyses included ChemoC node splitting and evidence certainty assessment (CINeMA).

**Results:**

Thirty-one RCTs and 34 single-arm studies (11,048 patients; 22 regimens) were included. Antibody-drug conjugate (ADC)-based therapies showed the most favorable efficacy overall. Sacituzumab govitecan (SG), sacituzumab tirumotecan (ST), and trastuzumab deruxtecan (T-DXd) ranked highest for ORR and PFS, with consistent benefits for SG and ST versus chemotherapy. T-DXd showed benefit mainly in HER2-low, HR-negative patients. For OS, SG and ST demonstrated clear advantages, while T-DXd showed non-significant effects. Combination chemotherapy outperformed single-agent chemotherapy for ORR and PFS. PARP inhibitors and PD-1–based regimens showed inconsistent efficacy across outcomes. Sensitivity analyses confirmed that incorporating single-arm data and separating chemotherapy nodes did not materially change treatment rankings or overall conclusions.

**Conclusion:**

ADCs, particularly SG and ST, were generally associated with favorable efficacy across outcomes in the subsequent-line treatment of advanced TNBC. These results may support clinical decision-making, although heterogeneity in study populations and the absence of direct comparisons highlight the need for further head-to-head trials.

## 1 Introduction

Triple-negative breast cancer (TNBC) is a subtype of breast cancer defined by the absence of estrogen receptor, progesterone receptor, and human epidermal growth factor receptor 2 expression. Compared with other breast cancer subtypes, TNBC is generally characterized by marked heterogeneity and relatively aggressive clinical behavior (1, 2). TNBC accounts for approximately 10%–20% of all breast cancer cases, tends to occur in younger women, and is associated with a higher likelihood of early recurrence and distant metastasis. Visceral organs such as the brain and lungs are common metastatic sites, contributing to the poor prognosis observed in this population (3, 4). As TNBC does not benefit from endocrine therapy or conventional targeted therapy, chemotherapy has long remained the mainstay of treatment. Commonly used agents include capecitabine, taxanes, anthracyclines, and platinum-based regimens. However, due to frequent chemoresistance, the clinical benefit of these treatments in the subsequent-line settings for advanced TNBC remains limited (5–7).

In recent years, several therapeutic advances have been made, including programmed cell death protein (PD-1) inhibitors, PARP inhibitors for patients with germline BRCA mutations, and antibody-drug conjugates (ADCs) targeting molecules such as TROP-2, thereby expanding treatment options beyond first-line therapy (8–11). Despite these developments, selecting an optimal regimen in clinical practice remains challenging. Most available studies compare a single novel agent with standard chemotherapy, such as sacituzumab govitecan (SG) versus chemotherapy or chemotherapy combined with immunotherapy versus chemotherapy alone (12, 13). However, head-to-head randomized controlled trials (RCTs) comparing different emerging therapeutic classes—such as various ADCs, immunotherapies, or targeted agents—are still limited. As a result, direct evidence to determine the relative efficacy of these regimens is currently insufficient.

Given the modest effectiveness of existing therapies and the increasing number of available treatment strategies for subsequent-line management of advanced TNBC, a comprehensive method that integrates evidence across studies and compares multiple regimens simultaneously is needed. Bayesian network meta-analysis (NMA) allows the synthesis of direct and indirect comparisons within a unified analytical framework, facilitating the estimation of relative treatment effects among numerous regimens with a shared comparator. By quantitatively ranking short-term and long-term efficacy outcomes across chemotherapy, immunotherapy, PARP inhibitors, and ADCs, this approach may provide useful evidence to support treatment decision-making for clinicians facing complex therapeutic choices.

## 2 Materials and methods

### 2.1 Data sources and study overview

This study was reported in accordance with the PRISMA 2020 guidelines and the PRISMA extension for NMA. Data were collected from RCTs evaluating different therapeutic regimens for advanced TNBC in the second-line or later setting, with the aim of comparing objective response rate (ORR), progression-free survival (PFS), and overall survival (OS).

### 2.2 Literature search and data extraction

A comprehensive literature search was performed in PubMed, Embase, Web of Science, and the Cochrane Library. Additional information regarding study results was retrieved from ClinicalTrials.gov. The search covered all records from database inception to September 30, 2025. The search strategy was based on database-specific free-text terms related to TNBC, randomized or clinical trials, and subsequent-line treatment, including terms such as “triple-negative breast cancer,” “TNBC,” “randomized controlled trial,” “clinical trial,” “second-line,” “third-line,” “later-line,” “subsequent therapy,” and “salvage therapy.” The strategy was adapted to the syntax of each database, and the complete database-specific search strategies are provided in Supplementary Text S1. Only studies published in English were considered. Reference lists of relevant articles were manually screened to identify additional eligible studies. Extracted information included first author, publication year, study design, baseline characteristics, treatment regimens, sample size in each group, and outcomes related to ORR, PFS, and OS. For time-to-event outcomes, hazard ratios (HRs) and 95% confidence intervals for PFS and OS were extracted from publications or ClinicalTrials.gov. When more than one HR was reported in a study (subgroup analyses), we selected the estimate corresponding to the second-line subgroup when available. To support the assessment of robustness, single-arm studies using regimens identical to those analyzed in the included RCTs were also identified and extracted.

### 2.3 Inclusion and exclusion criteria

Studies were included if they met the following criteria: (1) Histologically or cytologically confirmed TNBC; (2) Advanced TNBC with progression after at least one prior systemic therapy; (3) Prospective RCT design; (4) Reporting at least one of the outcomes ORR, PFS, or OS. Exclusion criteria were: (1) Basic science research, non-human studies, reviews, meta-analyses, or case reports; (2) Phase I clinical trials; (3) Insufficient clinical outcome data. Two researchers independently screened titles, abstracts, and full texts according to the criteria. Any disagreements were resolved through discussion with a third researcher. For studies including both first- and second-line populations, only those reporting separate second-line subgroup data were retained.

Risk of bias in RCTs was evaluated using the Cochrane Risk of Bias 2.0 tool, assessing randomization, allocation concealment, blinding, completeness of outcome data, selective reporting, and other potential biases. For non-randomized interventional studies, the ROBINS-I tool was applied.

### 2.4 Statistical analysis

#### 2.4.1 Bayesian network meta-analysis

The Bayesian NMA was performed using the gemtc package in R (version 4.5.0). ORR was analyzed using odds ratios (ORs), whereas HRs were used for PFS and OS. Given the potential variability across studies, random-effects models were used throughout. In several included trials, the control treatments consisted of physician’s choice single-agent chemotherapy regimens. These commonly involved capecitabine, gemcitabine, vinorelbine, docetaxel, paclitaxel, or eribulin, which were generally not reported separately. These agents were therefore grouped into a single node, “Single-agent Chemotherapy (ChemoS).” Similarly, combination chemotherapy arms—including irinotecan + capecitabine, gemcitabine + carboplatin, capecitabine + docetaxel, cisplatin + etoposide, vinflunine + capecitabine, vinorelbine + gemcitabine, and docetaxel + gemcitabine—were pooled into “Combination Chemotherapy (ChemoC).” The final analytical network comprised 22 distinct treatment regimens. Posterior distributions were estimated using Markov Chain Monte Carlo (MCMC) sampling. Adaptation was set at 10,000 iterations, followed by 50,000 sampling iterations with a thinning interval of 10. Convergence was assessed using the Gelman-Rubin diagnostic (R-hat <1.05), and chain stability was evaluated with the Geweke statistic (|Z| <1.96). Heterogeneity across studies was quantified using the I^2^ statistic, with values ≤ 25% considered low. Model fit was evaluated using the Deviance Information Criterion (DIC). Results were summarized using league tables and heat maps showing ORs or HRs with corresponding 95% credible intervals (CrIs) for all pairwise comparisons. The surface under the cumulative ranking curve (SUCRA) was used to rank treatments, with higher values reflecting more favorable performance. Forest plots comparing each regimen to reference treatments (ChemoS, ChemoC, and the pooled ADC class) were also generated to illustrate relative efficacy.

#### 2.4.2 Exploratory sensitivity analysis incorporating single-arm evidence

To test the robustness of the primary NMA, an exploratory sensitivity analysis was conducted by incorporating single-arm studies that evaluated regimens identical to those in the included RCTs. To prevent population overlap, duplicate reports, updated analyses, and subgroup publications were excluded, retaining only the most complete datasets. Single-arm evidence was strictly limited to treatments consistent with the RCT network. This analysis exclusively utilized ORR data. As a binary endpoint, ORR enables direct and reliable modeling using responder counts and sample sizes. To integrate single-arm data without overwhelming the primary RCT evidence, a down-weighted binomial likelihood approach was employed. A fractional weight (0.3) was applied to the sample sizes and responder counts of eligible single-arm studies, ensuring this data acted solely as weak supplementary information. Robustness was evaluated using a multidimensional assessment that included: (1) consistency in the direction of treatment effects (i.e., whether the 95% CrIs remained on the same side of the null before and after integration); (2) the proportional overlap of the 95% CrIs between the two models; and (3) the relative percentage change in the median point estimates.

#### 2.4.3 Sensitivity analysis of the pooled combination chemotherapy node

To assess the robustness of the pooled ChemoC node, it was separated into seven regimen-specific nodes corresponding to the treatments used in the included studies. The Bayesian NMA model was re-fitted, and key effect estimates for SG, Sacituzumab tirumotecan (ST), and trastuzumab deruxtecan (T-DXd) versus ChemoS were compared with the primary analysis to confirm that pooling did not materially affect the main findings.

#### 2.4.4 Certainty of evidence assessment using CINeMA

To evaluate the certainty of evidence for comparisons in the NMA, the CINeMA framework was applied. CINeMA assesses six domains: within-study bias, reporting bias, indirectness, imprecision, heterogeneity, and incoherence. For the outcomes of ORR, PFS, and OS, the assessment focused on clinically important comparisons involving the main treatment regimens identified in the network meta-analysis.

#### 2.4.5 Structured safety summary

To address the clinical relevance of treatment tolerability, we performed a structured safety summary. Safety outcomes were extracted from the included studies, including grade ≥3 adverse events (AEs), AE-related treatment discontinuation, AE-related dose reduction, grade ≥3 hematologic and gastrointestinal toxicities, grade ≥3 hepatic dysfunction, and interstitial lung disease (ILD)/pneumonitis. Given the heterogeneity and incomplete reporting of safety data across trials, a formal quantitative meta-analysis of safety outcomes was not performed. Instead, safety data were summarized descriptively by major treatment class. For each treatment class, the safety-evaluable population was calculated as the sum of patients included in the safety analysis across eligible studies. When multiple studies reported the same safety endpoint, a weighted average percentage was calculated using the safety-evaluable population as the denominator. The range of reported values and the number of contributing studies were provided to reflect between-study variability and data availability. Outcomes not reported in the original studies were recorded as not reported (NR).

## 3 Results

### 3.1 Study network characteristics and overview of included studies

**FIGURE 1:**
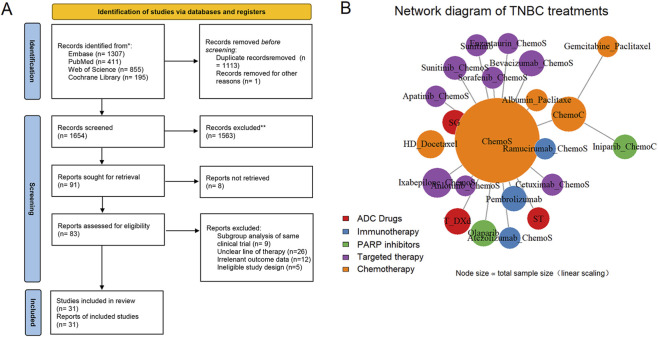
Study selection and network of eligible comparisons. **(A)** PRISMA flow diagram; **(B)** Network plot of treatment comparisons.

After the systematic literature screening, a total of 31 RCTs were included (12–42). The study selection process is summarized in Figure 1A. Study details and risk of bias assessment results are provided in Supplementary Table S1. An additional search identified 34 single-arm studies (some including subgroup data) that evaluated treatment regimens identical to those used in the included RCTs (43–76). Details of these studies and their risk of bias assessment resultsare provided in Supplementary Table S2. Altogether, 11,048 patients were included in this analysis, consisting of 8,283 patients from RCTs and 2,765 patients from single-arm studies. All patients receiving T-DXd in this analysis were from the HER2-low (IHC 1+ or IHC 2+/FISH-negative), hormone receptor (HR) -negative subgroup, with HR-positive patients excluded (36). All patients treated with olaparib harbored germline BRCA mutations. Potential effect modifiers across treatment categories are summarized in Supplementary Table S3. Overall, most treatment categories included patients receiving second-line or later-line therapy, and prior chemotherapy exposure was common across the network. However, several characteristics were unevenly distributed or incompletely reported. In particular, HER2-low status was mainly relevant to T-DXd-related evidence, whereas gBRCA mutation status was concentrated in studies of targeted agents such as olaparib. PD-L1 status, visceral metastases, prior immunotherapy, and prior ADC exposure were not consistently reported across all treatment categories.

### 3.2 Network structure and model quality assessment

The network structure of treatment comparisons is shown in Figure 1B. This graphical representation outlines the relationships among the included regimens. The resulting network demonstrated a multi-arm, star-shaped configuration centered around ChemoS, which served as the comparator in 29 direct comparisons. This structure reflects a moderately complex but well-connected evidence network. Following 10,000 adaptation iterations and 50,000 sampling iterations, all parameters achieved satisfactory convergence. The Gelman-Rubin diagnostic indicated R-hat values between 1.00 and 1.01, remaining below the commonly accepted threshold of 1.05, suggesting adequate mixing and convergence of the Markov chains. The Geweke diagnostic further supported convergence, with mean |Z| values below 1.96 across all parameters (Supplementary Text S2). Visual inspection of the trace plots (Supplementary Figure S1), density plots (Supplementary Figure S2), and Gelman-Rubin diagnostic plots (Supplementary Figure S3) also indicated stable chain mixing and satisfactory model convergence. Heterogeneity, as measured by the I^2^ statistic, was 13%, indicating a very low level of between-study variability.

### 3.3 Network meta-analysis for ORR

**FIGURE 2:**
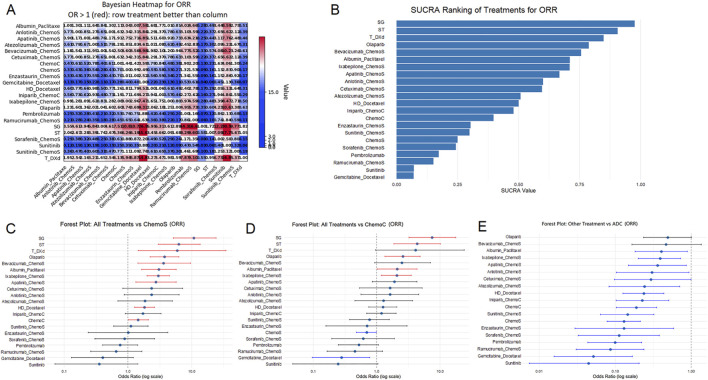
Network meta-analysis of objective response rate. **(A)** League heatmap of odds ratios, red areas (OR>1) indicate the row treatment is superior to the column treatment; **(B)** Ranking of treatments based on surface under the cumulative ranking curve values; **(C)** Forest plots of odds ratios for each treatment regimen compared to single-agent chemotherapy; **(D)** Forest plots of odds ratios for each treatment regimen compared to combination chemotherapy; **(E)** Forest plots of odds ratios for each treatment regimen compared to the pooled antibody-drug conjugate class.

The NMA of ORR generated a complete set of pairwise comparisons, which are summarized in the OR league table (Supplementary Table S4). An OR greater than 1 indicates that the row treatment is favored over the column treatment. A heatmap of these comparisons is presented in Figure 2A, with blue areas (OR < 1) representing relatively lower efficacy and red areas (OR > 1) representing relatively higher efficacy. In the SUCRA analysis, ADCs, including SG, ST, and T-DXd, tended to rank favorably for ORR (Figure 2B). Control arms consisting of single-agent chemotherapy, combination chemotherapy, and ADCs were pooled into ChemoS, ChemoC, and ADC, respectively. Head-to-head comparisons of all other treatments against these pooled groups are shown in Figures 2C–E. As shown in Figure 2C, ten treatments showed statistically significant ORR benefits compared with ChemoS. Among these, SG, ST, and T-DXd had favorable effect estimates and high SUCRA rankings. Figure 2D indicates that five regimens showed statistically significant ORR benefits compared with ChemoC. The top three regimens were again ADCs—SG and ST showed statistical significance, whereas T-DXd did not. In addition, ChemoS was inferior to ChemoC for ORR. Figure 2E further shows that the pooled ADC class exhibited significantly higher efficacy than most of the other 16 regimens; although two regimens showed a trend toward lower efficacy compared with ADCs, none reached statistical significance.

### 3.4 Network meta-analysis for PFS

**FIGURE 3:**
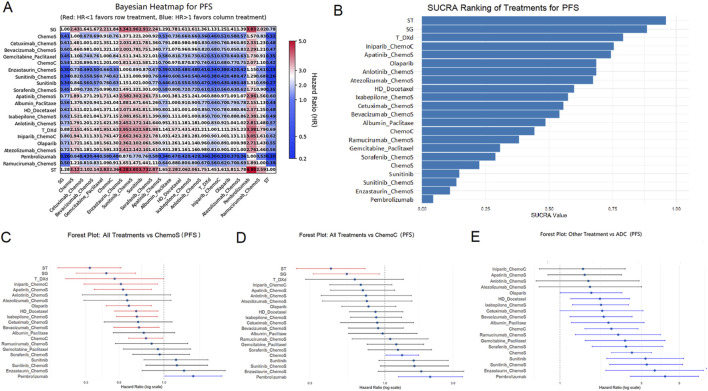
Network meta-analysis of progressionfree survival. **(A)** League heatmap of hazard ratio, blue areas (HR < 1) indicate the row treatment is superior to the column treatment; **(B)** Ranking of treatments based on surface under the cumulative ranking curve values; **(C)** Forest plots of hazard ratio for each treatment regimen compared to single-agent chemotherapy; **(D)** Forest plots of hazard ratio for each treatment regimen compared to combination chemotherapy; **(E)** Forest plots of hazard ratio for each treatment regimen compared to the pooled antibody-drug conjugate class.

PFS results, including HRs and 95% CrIs for all pairwise comparisons, are presented in the league table (Supplementary Table S5). An HR less than 1 favors the row treatment. Figure 3A displays these results in heatmap format, and the SUCRA-based ranking is shown in Figure 3B. Consistent with the ORR findings, the highest-ranked regimens were SG, ST, and T-DXd. Comparisons against the pooled ChemoS, ChemoC, and ADC groups are presented in Figures 3C–E. As shown in Figure 3C, ten regimens showed statistically significant PFS benefits compared with ChemoS, including SG, ST, and T-DXd. However, compared with ChemoC, statistically significant PFS benefits were observed only for SG and ST (Figure 3D). ChemoS remained significantly inferior to ChemoC in terms of PFS. As illustrated in Figure 3E, the pooled ADC class demonstrated significantly better PFS outcomes than 15 other regimens; four regimens showed a nonsignificant trend toward inferior efficacy relative to ADCs.

### 3.5 Network meta-analysis for OS

**FIGURE 4:**
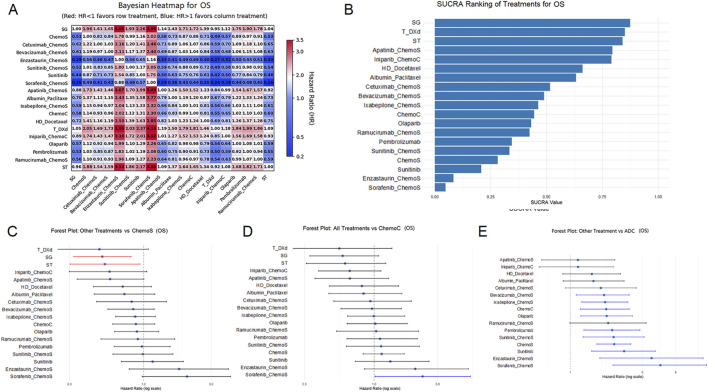
Network meta-analysis of overall survival **(A)**: League heatmap of hazard ratio, blue areas (HR < 1) indicate the row treatment is superior to the column treatment; **(B)** Ranking of treatments based on surface under the cumulative ranking curve values; **(C)** Forest plots of hazard ratio for each treatment regimen compared to single-agent chemotherapy; **(D)** Forest plots of hazard ratio for each treatment regimen compared to combination chemotherapy; **(E)** Forest plots of hazard ratio for each treatment regimen compared to the pooled antibody-drug conjugate class.

Because six RCTs did not report OS outcomes (18, 25, 30, 32, 37, 38), a separate OS network was constructed. Supplementary Figure S4 shows that this network remained connected. Pairwise comparisons based on OS are summarized in the league table (Supplementary Table S6), with HR < 1 favoring the row treatment. The corresponding heatmap is shown in Figure 4A, and SUCRA rankings are presented in Figure 4B. SG, T-DXd and ST remained the top three regimens for OS. Comparisons against ChemoS, ChemoC, and ADC are displayed in Figures 4C–E. As shown in Figure 4C, SG and ST consistently demonstrated statistically significant benefits compared with both ChemoS. As shown in Figure 4D, T-DXd, SG, and ST were the top three regimens compared with ChemoC, but none were statistically significant. Figure 4E shows that the pooled ADC class showed statistically significant OS benefits compared with ten other regimens. For six additional comparisons, the point estimates favored ADCs, but the 95% CrIs crossed the null, indicating that these differences were not statistically significant.

### 3.6 Exploratory sensitivity analysis incorporating single-arm studies

**FIGURE 5:**
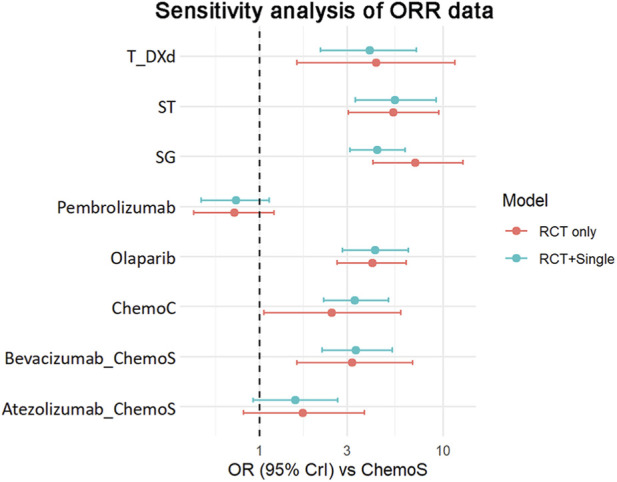
Comparison of treatment effect estimates before and after incorporating single-arm studies.

The multidimensional assessment of the sensitivity analysis revealed completely consistent effect directions across the evaluated treatments (Figure 5; Supplementary Table S7). Following the integration of single-arm data, no 95% CrIs crossed the null value differently, indicating that the fundamental clinical conclusions relative to ChemoS remained unaltered. For several treatments, including ST, pembrolizumab, and olaparib, the network estimates demonstrated high stability, with minimal changes in median point estimates (≤3.6%) and substantial CrI overlaps (>85%). Conversely, for certain regimens, integrating single-arm data resulted in a noticeable shift in the magnitude of the effect estimates. For example, the median OR for SG shifted from 7.050 (95% CrI: 4.148, 12.820) to 4.379 (95% CrI: 3.108, 6.215), reflecting a 37.9% relative reduction and a 21.3% CrI overlap. Moderate magnitude shifts were also observed for T-DXd and ChemoC. Despite these adjustments in point estimates, the overall statistical significance and direction of benefit were sustained across all evaluated treatments.

### 3.7 Sensitivity analysis for ChemoC node

**FIGURE 6:**
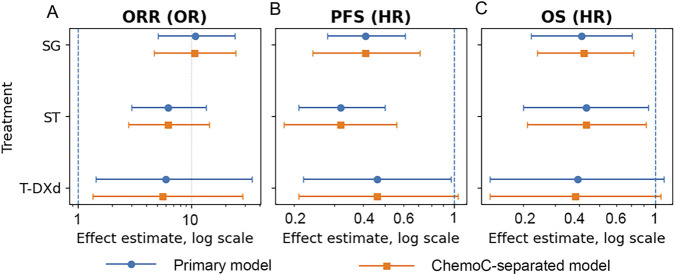
Sensitivity analysis after separating the ChemoC node. **(A)** objective response rate (odds ratio); **(B)** progression-free survival (hazard ratio); **(C)** overall survival (hazard ratio).

In the ChemoC-separated sensitivity analysis, five regimen-specific nodes were modeled (two studies were excluded due to network disconnection) (Supplementary Figure S5). The key treatment effects of SG, ST, and T-DXd versus ChemoS remained generally consistent with the primary model for ORR, PFS, and OS (Figure 6). The SUCRA rankings were broadly similar, with SG, ST, and T-DXd remaining among the top-ranked regimens, supporting the robustness of the main conclusions (Supplementary Figure S6).

### 3.8 Certainty assessment using CINeMA

**FIGURE 7:**
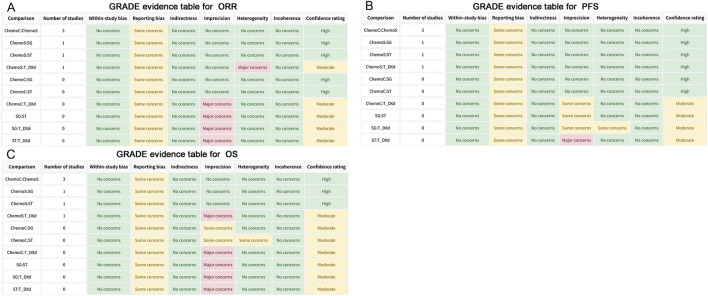
CINeMA assessment of evidence certainty for ORR, PFS, and OS in the network meta-analysis. **(A)** CINeMA assessment for ORR; **(B)** CINeMA assessment for PFS; **(C)** CINeMA assessment for OS.

The CINeMA assessment provided additional context for interpreting the network results. High confidence was observed for the comparisons of SG and ST versus ChemoS, as well as for ChemoC versus ChemoS across ORR, PFS, and OS (Figure 7; Supplementary Tables S8–S10). In contrast, evidence for T-DXd versus ChemoS was generally rated as moderate confidence, mainly driven by imprecision and, in some outcomes, heterogeneity or limited sample size in the HER2-low, HR-negative subgroup.

### 3.9 Structured safety summary

A structured summary of safety outcomes is presented in (Supplementary Table S11). Overall, adverse event reporting varied substantially across studies, and several safety endpoints were not consistently available across treatment classes. Among ADCs, the weighted incidence of grade ≥3 AEs was 58%, which is intermediate compared with other treatment classes. Occurrence of ILD/pneumonitis was primarily observed with ADCs and immunotherapy agents. Across other treatment classes, safety profiles were heterogeneous. Tyrosine kinase inhibitor (TKI)-based regimens and combination chemotherapy tended to show higher rates of severe toxicity or treatment modification, whereas immunotherapy-containing regimens had lower reported rates of grade ≥3 AEs, although reporting was less complete for several endpoints. Single-agent chemotherapy showed a broad but generally more moderate toxicity profile, with hematologic and gastrointestinal events remaining common.

## 4 Discussion

This Bayesian NMA quantitatively compared the relative efficacy of multiple treatment regimens for advanced TNBC in the second-line and subsequent-line settings. By constructing a multi-arm, star-shaped evidence network centered on standard chemotherapy, we were able to facilitate indirect comparisons across a wide range of regimens—including ADCs, immunotherapy, PARP inhibitors, and conventional chemotherapy—thereby helping to address the limited direct comparative data available in previous studies of advanced TNBC. An exploratory sensitivity analysis incorporating single-arm ORR evidence via a down-weighted binomial likelihood approach further supported the consistency of the primary RCT-based directional findings. Notably, the integration of this external data revealed distinct statistical impacts: for regimens such as T-DXd and ChemoC, the augmented sample size visibly improved the precision of the estimates by narrowing the 95% CrIs; whereas for regimens like SG, it resulted in a noticeable shift in the magnitude of the point estimate, yet the direction of effect and statistical significance remained unchanged across all evaluated treatments.

In this study, ADCs ranked highest for both ORR (a short-term endpoint) and PFS (a longer-term endpoint), with SG, ST, and T-DXd consistently comprising the top three regimens according to SUCRA. In indirect comparisons, SG and ST showed relatively consistent benefits compared with chemotherapy-based regimens, whereas T-DXd showed favorable estimates in several analyses but did not demonstrate statistically significant improvements in some studies due to the limited subgroup sample size and wider 95% CrIs. Nevertheless, these results are generally consistent with current trends in research on advanced TNBC (17, 77–79). SG and ST target TROP-2, a transmembrane protein widely expressed in epithelial tumors, including TNBC (80, 81). This expression pattern allows ADCs to deliver cytotoxic payloads more selectively to tumor cells while sparing normal tissues, a mechanism supported by several studies (82, 83). T-DXd targets HER2, and all patients treated with T-DXd in the included studies had HER2-low expression. HER2-low has recently emerged as a distinct biomarker category (84). For this subgroup, T-DXd has shown substantial benefit, and the pivotal trial by Modi et al. established its role as a standard treatment option in HER2-low advanced breast cancer (36). The ORR and PFS rankings observed in our analysis are consistent with these findings.

Because SUCRA rankings may be influenced by sparse and indirect evidence, we incorporated CINeMA assessment to place the ranking results in the context of evidence certainty. The high-confidence findings for SG and ST versus ChemoS, and for ChemoC versus ChemoS, strengthened the main conclusions, whereas the moderate-confidence evidence for T-DXd reflected remaining uncertainty in the HER2-low, HR-negative subgroup.

The interpretation of T-DXd results should be made in the context of the studied population. In our analysis, data for T-DXd were derived exclusively from the HER2-low, HR-negative subgroup of the DESTINY-Breast04 trial, which corresponds to HER2-low TNBC rather than HER2-zero TNBC. Therefore, the favorable effect estimates and SUCRA ranking of T-DXd observed in this network should be considered applicable only to patients with HER2-low disease, and these findings cannot be extrapolated to HER2-zero TNBC without further evidence. In addition, this subgroup included only 68 patients, which may have contributed to wider CrIs and to the absence of statistically significant differences between T-DXd and ChemoC across all efficacy endpoints (ORR, PFS, and OS), despite the favorable SUCRA ranking of T-DXd. Clinicians should therefore take individual HER2 expression status into account when weighing the relative benefits of T-DXd against other ADCs or chemotherapy.

Beyond HER2 and HR status, other biomarkers such as PD-L1 expression, gBRCA mutation, and prior treatment exposure may also act as effect modifiers influencing efficacy in the subsequent-line setting (85, 86). In this analysis, combination chemotherapy was associated with superiority over single-agent chemotherapy for both ORR and PFS, a finding that may hold practical relevance in regions where conventional chemotherapy remains widely used. By contrast, drugs such as the PARP inhibitor olaparib, the multitargeted agent anlotinib, and PD-1 inhibitors exhibited inconsistent results, showing advantages only in selected comparisons. These observations suggest that the efficacy of these agents may depend heavily on biomarker profiles or treatment history. Therefore, based on the currently available evidence, such treatments may not yet be considered priority options for an unselected population with advanced TNBC. Variability arising from patient heterogeneity is difficult to avoid in indirect comparisons based on aggregated study populations.

ORR and PFS results in this study were broadly consistent, suggesting that regimens producing meaningful tumor shrinkage may also be more likely to achieve durable disease control. However, the pattern observed for OS differed somewhat from that of ORR and PFS, with OS benefits appearing more limited overall. Compared with ChemoS, only SG and ST showed statistically significant OS benefits. Similarly, when compared with ChemoC, although T-DXd, SG, and ST ranked as the top three regimens, none reached statistical significance. Several factors may contribute to these discrepancies. OS is influenced by multiple variables, and missing OS data from six RCTs may have further reduced statistical power. In addition, OS typically requires longer follow-up and a greater number of events, while subsequent therapies—including ADCs administered after progression—may confound the true effect of the study treatment (87). Moreover, crossover designs used in some studies allowed patients in the control group to receive the investigational treatment after progression. Although ethically appropriate, such crossover may attenuate observed survival differences (88), making OS less sensitive to treatment-related effects. For T-DXd specifically, the lack of statistical significance may also be attributable to the wide CrIs resulting from the small sample size of the HER2-low, HR-negative subgroup.

The safety summary provides essential context for our efficacy findings. Although ADCs, particularly SG and ST, showed superior ORR and PFS, their distinct toxicity profiles require careful consideration of agent-specific risks: neutropenia and diarrhea for SG, hematologic toxicity for ST, and ILD for T-DXd. Therefore, favorable efficacy of ADCs does not imply uniform tolerability. Beyond ADCs, combination chemotherapy carries higher risks of severe neutropenia, while TKI-based regimens frequently lead to treatment discontinuation. Although immunotherapy reported fewer grade ≥3 adverse events, immune-related toxicities and incomplete reporting remain practical concerns. Ultimately, clinicians must balance efficacy with specific toxicities, biomarker status, prior treatments, comorbidities, and patient preference. Future prospective and real-world studies should standardize safety and quality-of-life reporting to better guide clinical decision-making.

Despite the methodological rigor applied, this study has several limitations. The most notable is the heterogeneity of included patient populations. Given the sparse, star-shaped structure of the network, SUCRA rankings should be considered supportive rather than definitive. Formal assessment of local inconsistency was not feasible. As a global check, we compared the consistency and unrelated mean effects models; the minimal ΔDIC indicated no meaningful improvement, but this result should be considered in the context of the network’s limited discriminatory power. Therefore, clinical interpretation should rely more on how large the differences are between treatments and how stable these estimates are across studies. To maintain network connectivity, patients with different biomarker profiles were included in the same analytical model. For example, all patients receiving T-DXd had HER2-low expression, excluding those with HER2-zero tumors. Conversely, all patients treated with olaparib carried gBRCA mutations. The validity of indirect comparisons in this NMA relies on the transitivity assumption, which may be influenced by patient and disease characteristics. Overall, most studies enrolled patients receiving second-line or later therapy with prior chemotherapy, supporting a basic level of comparability. Nevertheless, important differences in biomarker-defined populations, including HER2-low, gBRCA mutation, PD-L1 status, and prior exposure to ADCs or immunotherapy, suggest caution when comparing treatments across these subgroups. Additionally, multiple single-agent and combination chemotherapy regimens were pooled into ChemoS and ChemoC nodes to simplify the network. Although this approach is common in NMAs, it inevitably obscures potential differences among individual chemotherapy agents. For instance, eribulin and capecitabine may not be identical in efficacy, yet were aggregated due to inability to separate their data. Our exploratory sensitivity analysis separating combination chemotherapy regimens into regimen-specific nodes confirmed that the main efficacy conclusions were not materially affected. This further suggests that pooling combination chemotherapy regimens in the primary analysis did not materially influence the main findings. Furthermore, although we initially intended to perform a safety analysis, heterogeneity in adverse event reporting formats and grading scales across studies precluded unified synthesis. Consequently, our conclusions focus on efficacy, and clinical decision-making should integrate both our efficacy findings and safety results from individual studies.

Several directions for future research can be considered. Head-to-head RCTs comparing different ADCs would be highly informative in clarifying relative efficacy and safety. Further research on predictive biomarkers may help refine individualized treatment strategies, such as identifying molecular markers that predict response to specific targeted or immunotherapeutic agents. In addition, real-world studies involving broader and more heterogeneous patient populations would be valuable for validating the findings of this NMA and complementing clinical trial data, particularly with respect to long-term safety and quality-of-life outcomes.

## 5 Conclusion

In conclusion, this Bayesian NMA suggests that, among available subsequent-line treatments for advanced TNBC, ADCs—particularly the TROP-2–targeting agents SG and ST—showed relatively consistent and higher efficacy across multiple endpoints. For patients with HER2-low expression, T-DXd appears to be an effective option. The finding that combination chemotherapy outperforms single-agent chemotherapy was consistently observed across endpoints for both short-term and long-term outcomes. Although this study is limited by population heterogeneity, variation in evidence availability, and differences in indicated treatment populations, the results may still offer useful guidance for clinicians when considering subsequent-line therapeutic options. Continued generation of direct comparative evidence will be important for further refining treatment strategies.

## Data Availability

The datasets presented in this study can be found in online repositories. The names of the repository/repositories and accession number(s) can be found in the article/Supplementary Material.
