# Supplementary figures and images for: Comparative efficacy of subsequent-line therapies for advanced triple-negative breast cancer: a bayesian network meta-analysis
Source: Oncol Rev. 2026 Jul 9;20:1834466. doi: 10.3389/or.2026.1834466 (PMC13391881; doi:10.3389/or.2026.1834466)

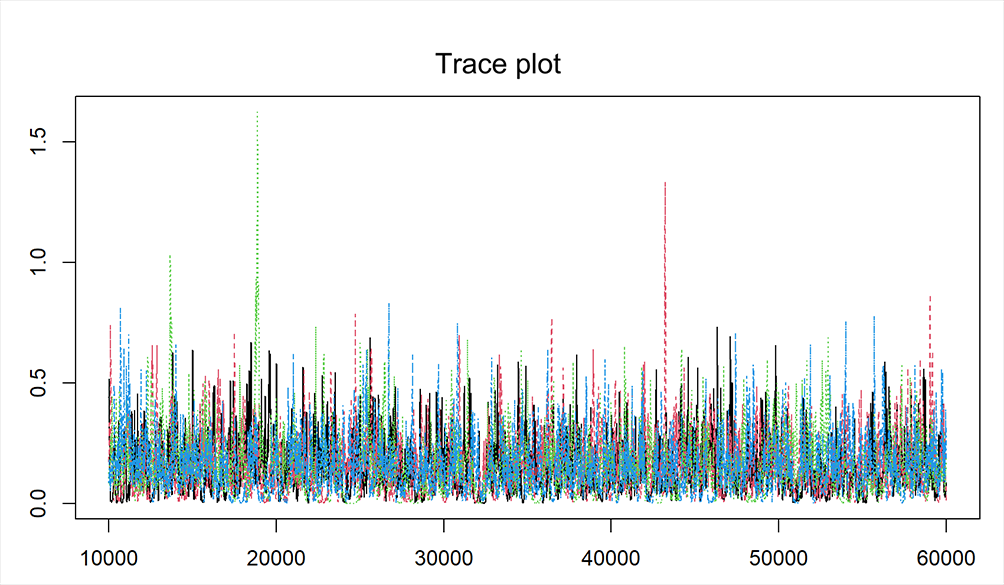

Supplement: Supplementary file 2 [file Image1.TIFF]

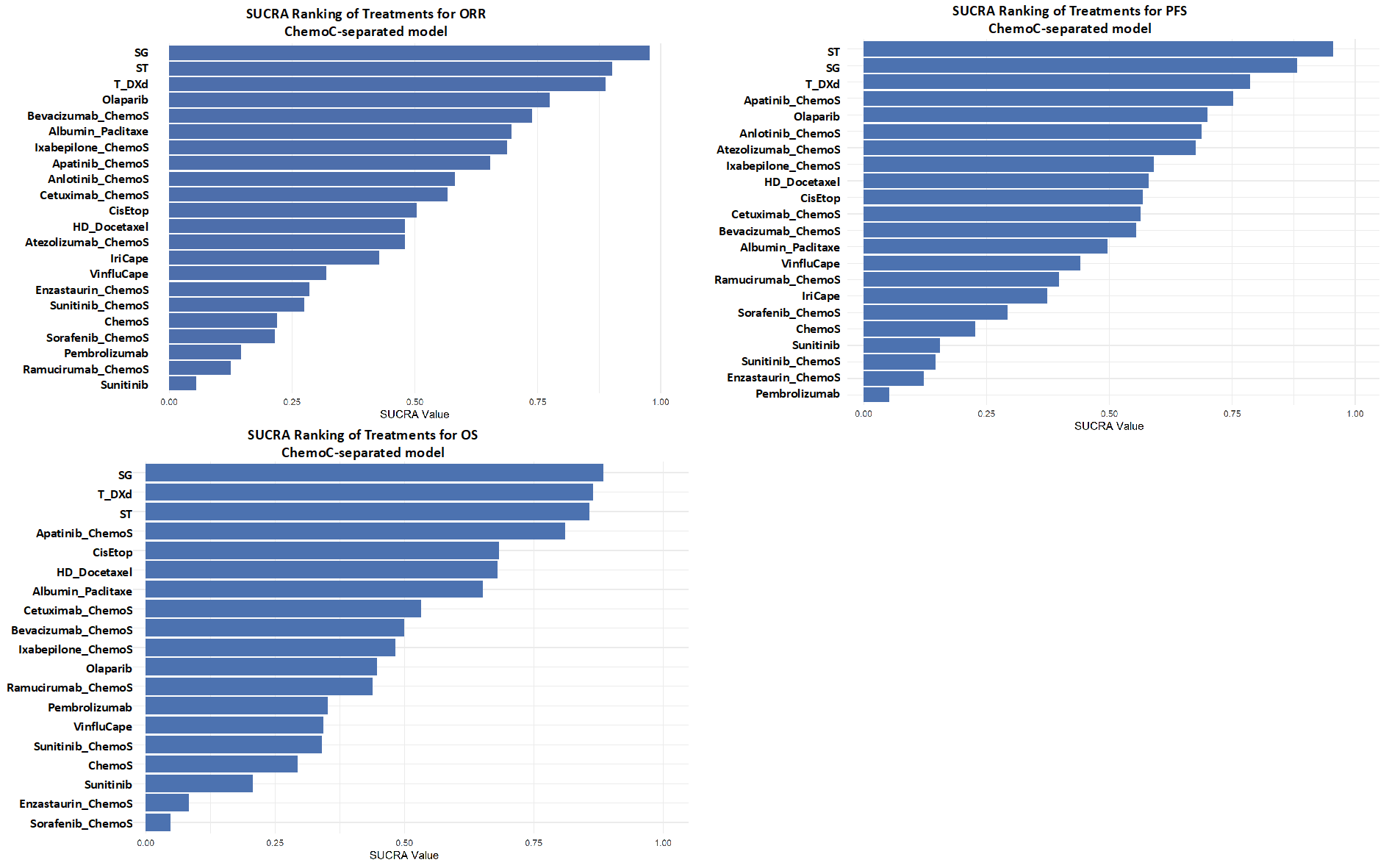

Supplement: Supplementary file 4 [file Image6.TIF]

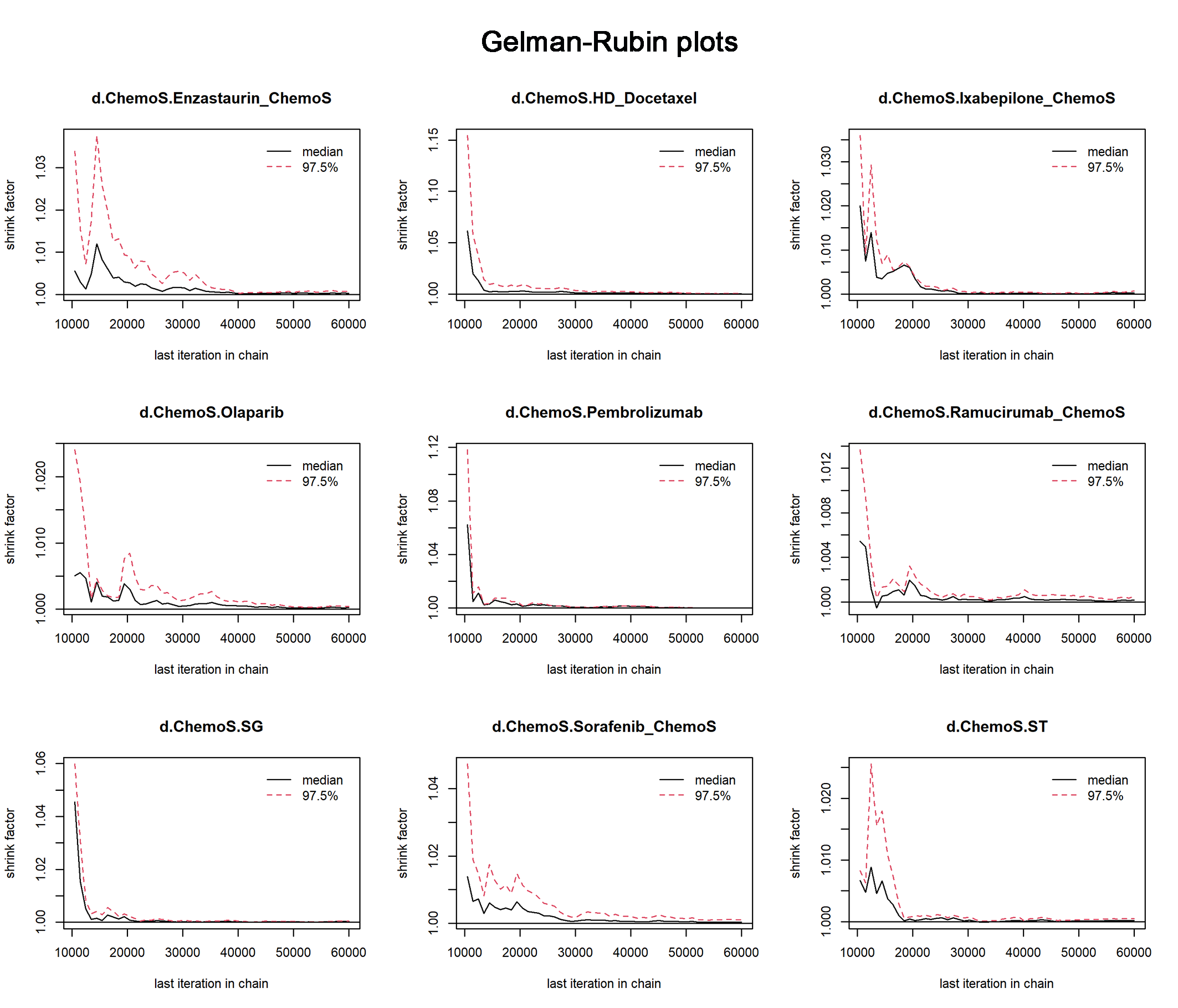

Supplement: Supplementary file 7 [file Image3.TIF]

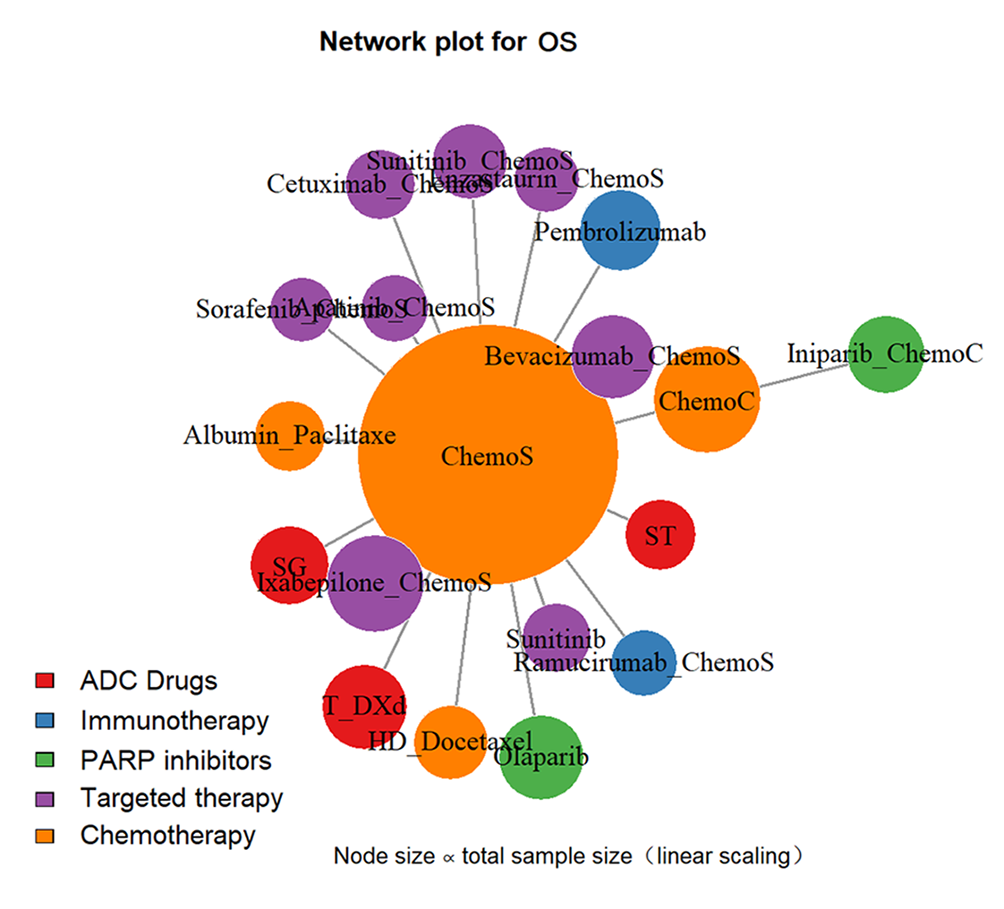

Supplement: Supplementary file 8 [file Image4.TIF]

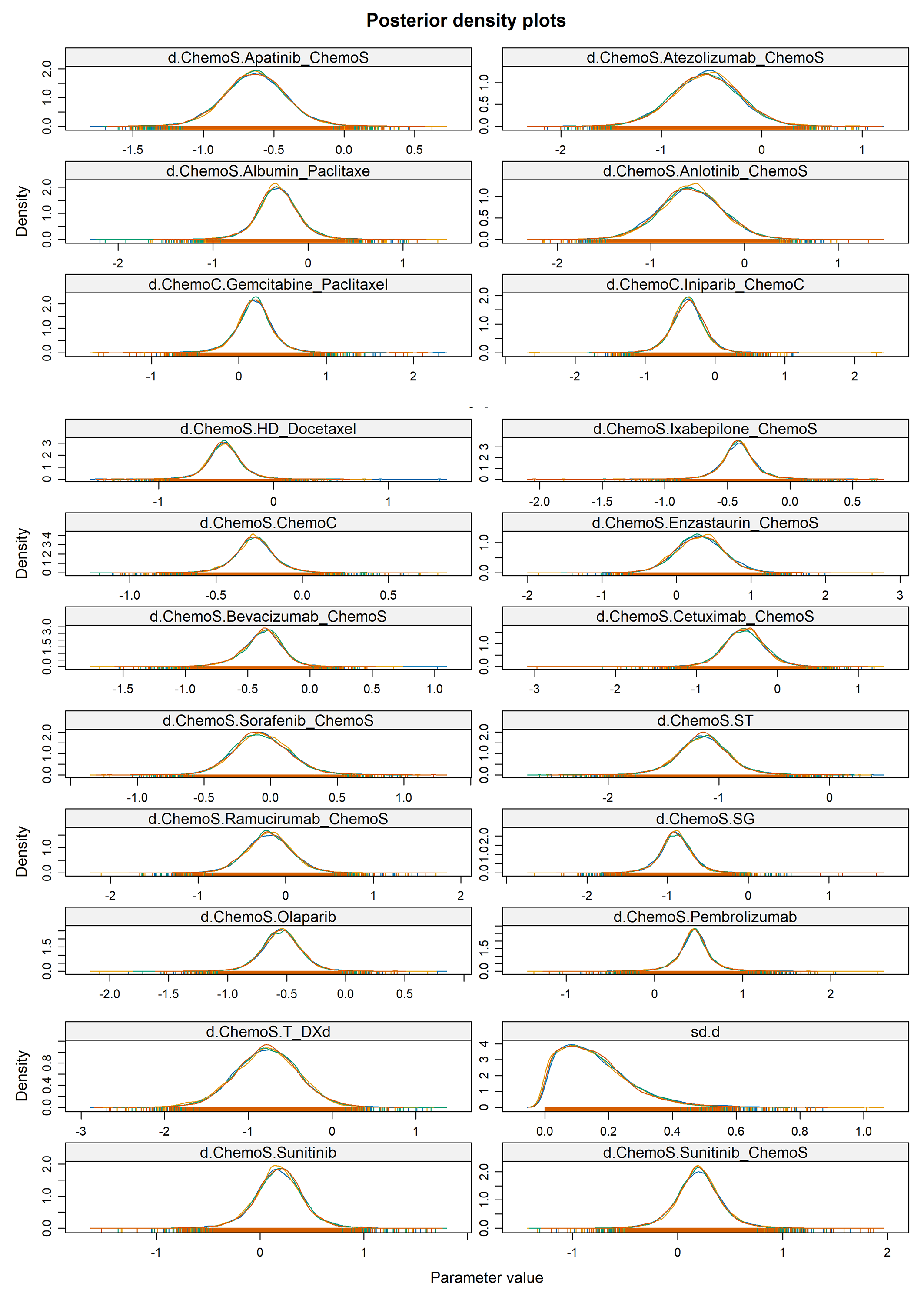

Supplement: Supplementary file 9 [file Image2.TIF]

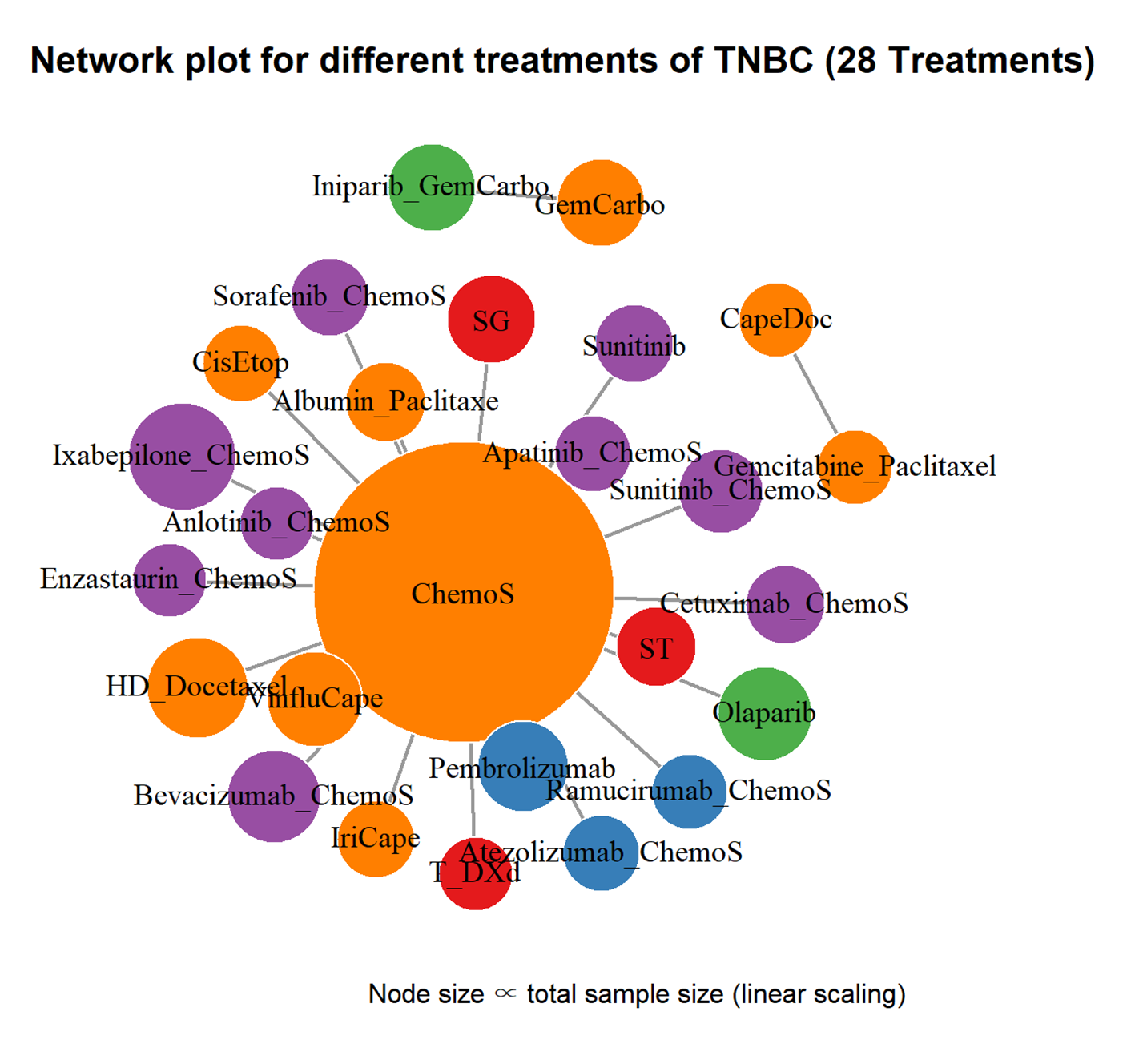

Supplement: Supplementary file 16 [file Image5.TIF]
