# Supplementary material for: Comparative efficacy of subsequent-line therapies for advanced triple-negative breast cancer: a bayesian network meta-analysis
Source: Oncol Rev. 2026 Jul 9;20:1834466. doi: 10.3389/or.2026.1834466 (PMC13391881; doi:10.3389/or.2026.1834466)
Supplement: Supplementary file 5 [file Supplementaryfile1.docx]

**Supplementary Text 1. Full search strategies**

**Database: PubMed**

Time span: Inception to September 30, 2025

Language restriction: English

("triple negative breast cancer" OR "TNBC" OR "triple-negative breast carcinoma" OR "triple-negative breast neoplasm") AND ("advanced" OR "metastatic" OR "recurrent" OR "second-line" OR "third-line" OR "later-line" OR "subsequent therapy" OR "salvage therapy") AND ("randomized controlled trial" OR "randomised controlled trial" OR "clinical trial")

**Database: Embase**

Time span: Inception to September 30, 2025

Language restriction: English

('triple negative breast cancer' OR 'TNBC' OR 'triple-negative breast carcinoma' OR 'triple-negative breast neoplasm') AND ('advanced' OR 'metastatic' OR 'recurrent' OR 'second-line' OR 'third-line' OR 'later-line' OR 'subsequent therapy' OR 'salvage therapy') AND ('randomized controlled trial' OR 'randomised controlled trial' OR 'clinical trial')

**Database: Web of Science**

Time span: Inception to September 30, 2025

Language restriction: English

TS=("triple negative breast cancer" OR "TNBC" OR "triple-negative breast carcinoma" OR "triple-negative breast neoplasm") AND TS=("advanced" OR "metastatic" OR "recurrent" OR "second-line" OR "third-line" OR "later-line" OR "subsequent therapy" OR "salvage therapy") AND TS=("randomized controlled trial" OR "randomised controlled trial" OR "clinical trial")

**Database: Cochrane Library**

Time span: Inception to September 30, 2025

Language restriction: English

("triple negative breast cancer" OR "TNBC" OR "triple-negative breast carcinoma" OR "triple-negative breast neoplasm") AND ("advanced" OR "metastatic" OR "recurrent" OR "second-line" OR "third-line" OR "later-line" OR "subsequent therapy" OR "salvage therapy") AND ("randomized controlled trial" OR "randomised controlled trial" OR "clinical trial")
