# Supplementary material for: Comparative efficacy of subsequent-line therapies for advanced triple-negative breast cancer: a bayesian network meta-analysis
Source: Oncol Rev. 2026 Jul 9;20:1834466. doi: 10.3389/or.2026.1834466 (PMC13391881; doi:10.3389/or.2026.1834466)
Supplement: Supplementary file 14 [file Supplementaryfile2.docx]

=== Gelman diagnostic（R-hat）===

Potential scale reduction factors:

Point est.

d.ChemoC.Gemcitabine_Paclitaxel 1

d.ChemoC.Iniparib_ChemoC 1

d.ChemoS.Albumin_Paclitaxe 1

d.ChemoS.Anlotinib_ChemoS 1

d.ChemoS.Apatinib_ChemoS 1

d.ChemoS.Atezolizumab_ChemoS 1

d.ChemoS.Bevacizumab_ChemoS 1

d.ChemoS.Cetuximab_ChemoS 1

d.ChemoS.ChemoC 1

d.ChemoS.Enzastaurin_ChemoS 1

d.ChemoS.HD_Docetaxel 1

d.ChemoS.Ixabepilone_ChemoS 1

d.ChemoS.Olaparib 1

d.ChemoS.Pembrolizumab 1

d.ChemoS.Ramucirumab_ChemoS 1

d.ChemoS.SG 1

d.ChemoS.Sorafenib_ChemoS 1

d.ChemoS.ST 1

d.ChemoS.Sunitinib 1

d.ChemoS.Sunitinib_ChemoS 1

d.ChemoS.T_DXd 1

sd.d 1

Upper C.I.

d.ChemoC.Gemcitabine_Paclitaxel 1.00

d.ChemoC.Iniparib_ChemoC 1.00

d.ChemoS.Albumin_Paclitaxe 1.00

d.ChemoS.Anlotinib_ChemoS 1.00

d.ChemoS.Apatinib_ChemoS 1.01

d.ChemoS.Atezolizumab_ChemoS 1.00

d.ChemoS.Bevacizumab_ChemoS 1.00

d.ChemoS.Cetuximab_ChemoS 1.00

d.ChemoS.ChemoC 1.00

d.ChemoS.Enzastaurin_ChemoS 1.00

d.ChemoS.HD_Docetaxel 1.00

d.ChemoS.Ixabepilone_ChemoS 1.00

d.ChemoS.Olaparib 1.00

d.ChemoS.Pembrolizumab 1.00

d.ChemoS.Ramucirumab_ChemoS 1.00

d.ChemoS.SG 1.00

d.ChemoS.Sorafenib_ChemoS 1.00

d.ChemoS.ST 1.00

d.ChemoS.Sunitinib 1.00

d.ChemoS.Sunitinib_ChemoS 1.00

d.ChemoS.T_DXd 1.00

sd.d 1.01

All parameters had R-hat ≤ 1.1, indicating good model convergence.

> # Geweke diagnostic

> cat("\n=== Geweke diagnostic (Z-scores)===\n")

=== Geweke diagnostic (Z-scores)===

> geweke_result <- geweke.diag(mcmc_chains)

> print(geweke_result)

[[1]]

Fraction in 1st window = 0.1

Fraction in 2nd window = 0.5

d.ChemoC.Gemcitabine_Paclitaxel

1.317115

d.ChemoC.Iniparib_ChemoC

-0.402321

d.ChemoS.Albumin_Paclitaxe

0.534221

d.ChemoS.Anlotinib_ChemoS

0.610253

d.ChemoS.Apatinib_ChemoS

-0.717646

d.ChemoS.Atezolizumab_ChemoS

-0.840890

d.ChemoS.Bevacizumab_ChemoS

-2.754383

d.ChemoS.Cetuximab_ChemoS

0.797143

d.ChemoS.ChemoC

-0.063197

d.ChemoS.Enzastaurin_ChemoS

-0.943738

d.ChemoS.HD_Docetaxel

-0.420798

d.ChemoS.Ixabepilone_ChemoS

0.513207

d.ChemoS.Olaparib

-0.259196

d.ChemoS.Pembrolizumab

-0.005788

d.ChemoS.Ramucirumab_ChemoS

-0.207248

d.ChemoS.SG

0.346937

d.ChemoS.Sorafenib_ChemoS

1.364093

d.ChemoS.ST

0.013078

d.ChemoS.Sunitinib

0.769217

d.ChemoS.Sunitinib_ChemoS

0.610032

d.ChemoS.T_DXd

0.862958

sd.d

1.465332

[[2]]

Fraction in 1st window = 0.1

Fraction in 2nd window = 0.5

d.ChemoC.Gemcitabine_Paclitaxel

1.95083

d.ChemoC.Iniparib_ChemoC

-0.75126

d.ChemoS.Albumin_Paclitaxe

-0.64224

d.ChemoS.Anlotinib_ChemoS

-1.22835

d.ChemoS.Apatinib_ChemoS

-0.21008

d.ChemoS.Atezolizumab_ChemoS

0.28426

d.ChemoS.Bevacizumab_ChemoS

0.45284

d.ChemoS.Cetuximab_ChemoS

2.90157

d.ChemoS.ChemoC

1.77486

d.ChemoS.Enzastaurin_ChemoS

-0.92196

d.ChemoS.HD_Docetaxel

0.29598

d.ChemoS.Ixabepilone_ChemoS

0.09895

d.ChemoS.Olaparib

0.29622

d.ChemoS.Pembrolizumab

-0.14451

d.ChemoS.Ramucirumab_ChemoS

0.46870

d.ChemoS.SG

-0.57018

d.ChemoS.Sorafenib_ChemoS

1.61582

d.ChemoS.ST

0.07488

d.ChemoS.Sunitinib

-0.31365

d.ChemoS.Sunitinib_ChemoS

1.27417

d.ChemoS.T_DXd

0.73572

sd.d

0.38795

[[3]]

Fraction in 1st window = 0.1

Fraction in 2nd window = 0.5

d.ChemoC.Gemcitabine_Paclitaxel

1.93648

d.ChemoC.Iniparib_ChemoC

-0.84375

d.ChemoS.Albumin_Paclitaxe

-1.30838

d.ChemoS.Anlotinib_ChemoS

0.12500

d.ChemoS.Apatinib_ChemoS

-0.71037

d.ChemoS.Atezolizumab_ChemoS

-2.38949

d.ChemoS.Bevacizumab_ChemoS

2.83315

d.ChemoS.Cetuximab_ChemoS

0.57189

d.ChemoS.ChemoC

0.61695

d.ChemoS.Enzastaurin_ChemoS

0.62049

d.ChemoS.HD_Docetaxel

-3.68020

d.ChemoS.Ixabepilone_ChemoS

0.53590

d.ChemoS.Olaparib

1.11761

d.ChemoS.Pembrolizumab

-1.86810

d.ChemoS.Ramucirumab_ChemoS

-0.88974

d.ChemoS.SG

-0.05561

d.ChemoS.Sorafenib_ChemoS

-0.35251

d.ChemoS.ST

-1.51906

d.ChemoS.Sunitinib

0.83072

d.ChemoS.Sunitinib_ChemoS

-0.33088

d.ChemoS.T_DXd

2.54820

sd.d

-2.41846

[[4]]

Fraction in 1st window = 0.1

Fraction in 2nd window = 0.5

d.ChemoC.Gemcitabine_Paclitaxel

0.1784

d.ChemoC.Iniparib_ChemoC

0.7081

d.ChemoS.Albumin_Paclitaxe

0.9710

d.ChemoS.Anlotinib_ChemoS

1.1668

d.ChemoS.Apatinib_ChemoS

1.7172

d.ChemoS.Atezolizumab_ChemoS

0.8193

d.ChemoS.Bevacizumab_ChemoS

0.4458

d.ChemoS.Cetuximab_ChemoS

2.9419

d.ChemoS.ChemoC

1.4651

d.ChemoS.Enzastaurin_ChemoS

2.1100

d.ChemoS.HD_Docetaxel

0.8041

d.ChemoS.Ixabepilone_ChemoS

1.2287

d.ChemoS.Olaparib

0.3206

d.ChemoS.Pembrolizumab

-0.3471

d.ChemoS.Ramucirumab_ChemoS

0.1257

d.ChemoS.SG

-0.6630

d.ChemoS.Sorafenib_ChemoS

-1.1503

d.ChemoS.ST

-1.5373

d.ChemoS.Sunitinib

0.7821

d.ChemoS.Sunitinib_ChemoS

-1.6242

d.ChemoS.T_DXd

-1.7436

sd.d

-0.5976

-- Model fit (residual deviance):

Dbar pD DIC

32.301 25.484 58.020

29 data points, ratio 1.114, I^2 = 13%
